# Metagenomic Next-Generation Sequencing as a Robust Diagnostic Approach for Human *Orf* Virus Infection Undetectable by Conventional Microbiological Methods

**DOI:** 10.1093/ofid/ofag724

**Published:** 2026-11-13

**Authors:** Jiaao Wang, Rong Wang, Yuyao Yin, Jin Cheng, Yue Wang, Si Liu, Xiaoyang Zhang, Shangyu Tu, Yifan Guo, Qi Wang, Hui Wang, Hongbin Chen

**Affiliations:** Department of Clinical Laboratory, Peking University People's Hospital, Beijing, China; Department of Clinical Laboratory Medicine, Xiangyang Central Hospital, Affiliated Hospital of Hubei University of Arts and Science, Xiangyang, China; Department of Clinical Laboratory, Peking University People's Hospital, Beijing, China; Department of Radiology, Peking University People's Hospital, Beijing, China; Department of Infectious Diseases, Peking University People's Hospital, Beijing, China; Department of Clinical Laboratory, Peking University People's Hospital, Beijing, China; Department of Clinical Laboratory, Peking University People's Hospital, Beijing, China; Department of Clinical Laboratory, Peking University People's Hospital, Beijing, China; Department of Clinical Laboratory, Peking University People's Hospital, Beijing, China; Department of Clinical Laboratory, Peking University People's Hospital, Beijing, China; Department of Clinical Laboratory, Peking University People's Hospital, Beijing, China; Department of Clinical Laboratory, Peking University People's Hospital, Beijing, China

**Keywords:** metagenomic next-generation sequencing, molecular diagnosis, *orf* virus, *parapoxvirus*, zoonotic infection

## Abstract

**Background:**

*Orf* virus (ORFV) is a significant pathogen known for its highly contagious nature. This study reports the case of a 55-year-old male farmer who developed fever and wound infection following an untreated sheep bite. The hospital utilized metagenomic next-generation sequencing (mNGS) technology to identify the rare pathogen—*orf* virus. Since *orf* virus cannot be detected through conventional bacterial and fungal cultures, this study underscores the importance of mNGS in identifying rare pathogens.

**Method:**

The patient developed fever and wound suppuration on day 11 following a sheep-related injury and was admitted to our hospital on day 17 because of persistent intermittent fever despite prior antibiotic treatment. Magnetic resonance imaging revealed soft tissue damage. Blood cultures, Gram and Ziehl–Neelsen staining, and bacterial and fungal cultures of wound secretions yielded negative results. Subsequently, mNGS detected *orf* virus in the wound secretion specimen. To verify the mNGS results, a quantitative polymerase chain reaction (qPCR) analysis targeting the *B2L* gene of parapoxvirus was performed on the wound secretion specimen.

**Results:**

mNGS identified ORFV in the wound secretion specimen. *B2L*-targeted qPCR performed on the same specimen yielded a positive result, while Sanger sequencing provided limited additional sequence-level support consistent with a parapoxvirus *B2L* target.

**Conclusions:**

*Orf* virus cannot be cultured in most diagnostic laboratory cell lines, and there is no routine clinical detection for *orf* virus. Therefore, mNGS emerges as a powerful tool for detecting such infections. This case demonstrates that mNGS provides an effective diagnostic strategy for infections that cannot be identified by traditional culture methods.

*Orf* virus (ORFV) is a significant pathogen and a highly contagious virus. It is responsible for causing orf, also known as ecthyma contagiosum, a highly contagious dermatological disease [1]. Among animal poxviruses, *orf* virus is one of the most extensively studied viruses, next to *vaccinia* virus, owing to its worldwide distribution and zoonotic significance [2]. The virus primarily affects sheep and goats [3], but it can also infect camels, cervids (such as deer and reindeer), other ruminants (such as musk oxen), as well as dogs, cats, and squirrels [4–8]. The virus also has zoonotic potential, posing a greater risk to people who handle animals, such as farmers, animal caregivers, and veterinarians [9]. The virus can be transmitted to humans through contact with infected animals, such as sheep, goats, and camels [10, 11]. Human-to-human transmission can occur occasionally, but it is extremely rare [12].

In infected animals, the virus manifests as proliferative lesions on the skin or mucous membranes, known as infectious dermatitis or contagious pustular dermatitis. These lesions commonly appear on the lips, oral cavity, ears, eyelids, and nostrils of the infected animals and are less frequently found on the udder, genitalia, and feet [13, 14]. In humans, the lesions typically occur on exposed skin areas that come into contact with infected animals, particularly on the hands, fingers, and forearms, and can present as polymorphic erythema [15–17]. The course of an *orf* virus infection is typically benign, with complete healing occurring in approximately 4–6 weeks [16]. In countries with Muslim populations, outbreaks of *orf* virus hand infection occur annually during the Eid al-Adha festival. During the festival, thousands of sheep are slaughtered by nonprofessionals, and hand injuries are unavoidable during the slaughtering process, leading many people to become infected with the *orf* virus [18, 19].

*Orf* virus belongs to the *Poxviridae* family and the *Parapoxvirus* genus. The viral nucleic acid of *orf* virus is composed of linear double-stranded DNA, approximately 134 to 140 kb in length, encoding about 132 genes [20]. The genome is structurally and functionally divided into 2 parts: one part is the central region, consisting of genes that primarily encode factors necessary for viral DNA replication and transcription; the other part consists of 2 inverted terminal repeat sequences, occupying about 20% of the terminal ends of the viral genome, and encodes factors related to virulence, pathogenesis, immune evasion/regulation, and host range [21, 22]. These include IL-10-like genes, vascular endothelial growth factor, granulocyte–macrophage colony-stimulating factor/interleukin-2 inhibitory factor, apoptosis inhibitors, and interferon resistance genes [20]. The envelope gene (*B2L*) encodes a highly immunogenic envelope protein, which is used for molecular diagnosis, characterization, and phylogenetic studies [23, 24].

Various diagnostic methods are currently available for ORFV detection. Virus isolation remains the gold standard for ORFV detection, although this method is labor-intensive, time-consuming, and relatively insensitive. Indirect enzyme-linked immunosorbent assay is relatively easy to perform, inexpensive, and potentially suitable for high-throughput testing, making it the most widely used serological diagnostic method. In patients with a relevant history of animal exposure but atypical or nonspecific skin lesions, histopathological examination of biopsy specimens or PCR testing may help establish the diagnosis [25].

In this study, we report a case of human ORFV infection following a sheep-related finger injury. Conventional microbiological examinations did not identify the causative pathogen. Metagenomic next-generation sequencing (mNGS) identified ORFV in a wound secretion specimen, with additional molecular support from *B2L*-targeted quantitative polymerase chain reaction (qPCR). The findings were consistent with the patient's exposure history and clinical presentation, suggesting that transmission likely occurred during the sheep-related injury.

## METHODS

### Case Information Collection

This study included a 55-year-old male farmer, and the collection of clinical data was approved by the Institutional Review Board of Peking University People's Hospital. The day of the sheep-related injury was designated as day 0. The patient developed fever and wound suppuration on day 11 and was admitted to the Infectious Diseases Department of our hospital on day 17 because of persistent intermittent fever despite previous antibiotic treatment. Clinical information was collected before and during hospitalization, as described below:

1. Medical history: Including the patient's personal history, medical history, allergy history, family history, and the occurrence and development process of this illness.
2. Clinical data: Including physical examination, wound assessment, recording of clinical symptoms, and the medications and treatment methods used.
3. Laboratory tests: Complete blood count and biochemical indices.
4. Microbiological testing: Pathogen detection, such as bacterial culture and fungal culture. mNGS technology was used to detect pathogens in the patient's pus, and qPCR technology was used to confirm certain pathogens.
5. Imaging data: The patient underwent magnetic resonance imaging (MRI) after admission to assess soft tissue damage.
6. Treatment response records: Documenting the baseline status of the patient before antibiotic treatment, clinical changes during the treatment process, and follow-up after treatment completion.

### Conventional Microbial Culture

For blood samples, blood cultures were first conducted using blood culture bottles. After the blood culture bottles tested positive, the samples were inoculated onto 1 blood agar plate and 1 MacConkey agar plate. For anaerobic bottles that tested positive, 2 blood agar plates and 1 MacConkey agar plate were inoculated, with 1 blood agar plate placed in an anaerobic bag for incubation.

For pus samples, bacterial cultures can be directly inoculated onto blood agar plates, MacConkey agar plates, and chocolate agar plates, while fungal cultures were inoculated onto Sabouraud dextrose agar plates.

### DNA Extraction, Library Preparation, and Sequencing

DNA was extracted from wound secretion samples using the QIAamp DNA Microbiome Kit (Qiagen) according to the manufacturer's instructions. Human DNA was removed using Benzonase (Qiagen). Sterile deionized water was extracted alongside the specimens as a negative control. DNA concentrations were determined using the Qubit dsDNA HS (high sensitivity) Assay Kit (Thermo Fisher Scientific). Sequencing libraries were prepared using the TruePrep™ DNA Library Prep Kit (Vazyme), and sequencing was performed on the Illumina NextSeq 550 platform using 75-bp single-end reads.

### Bioinformatics Analysis of Sequencing Data

Raw sequencing data were processed by removing adapter-contaminated, low-quality, low-complexity, and short reads using Trimmomatic (v0.39) [26]. After quality control, reads were aligned to the human reference genome GRCh38 (hg38) using Bowtie2 (v2.3.5.1) [27], and host-derived reads were removed. The remaining nonhuman reads were subjected to taxonomic classification using Kraken2 (v2.0.8-beta) [28]. A custom microbial reference database was constructed from genome sequences downloaded from the National Center for Biotechnology Information (NCBI), including 185 630 bacterial genomes, 308 archaeal genomes, 54 fungal genomes, 9021 viral genomes, 178 invertebrate genomes, 39 protist genomes, and the human reference genome (hg38). The database used for analysis in this study was the validated in-house database version routinely implemented in our laboratory workflow, which was based on NCBI genomes available as of May 2019 [29]. To ensure consistency of the bioinformatic pipeline and comparability of clinical testing results within our laboratory, the same locked database version was used for this case.

The sample generated approximately 14.12 million quality-filtered reads. Candidate microorganisms identified by Kraken2 were interpreted in conjunction with the negative control and clinical context.

### Clinical Reporting Criteria for mNGS Test

The clinical positive reporting criteria for mNGS utilized in this study were as follows. For viruses, the criterion was RPM (reads per million) ratio ≥3.0; for bacteria and fungi, the criterion was RPM ratio ≥10.0 (with reads ≥1 applied for nontuberculous mycobacteria, *Legionella*, etc.) [30–33]. For microorganisms that do not meet the positive reporting threshold, after discussion by clinical physicians, microorganisms considered clinically relevant were reported as “possible.”

### *B2L*-Targeted qPCR and Sanger Sequencing

To validate the mNGS findings, qPCR targeting the *B2L* gene of parapoxviruses was performed using the same wound secretion specimen subjected to mNGS. The assay was conducted using a previously published primer and probe set described by Andreani et al [34]. The sequences were as follows: forward primer (B2L-F), 5′-CGGTGCAGCACGAGGTC-3′; reverse primer (B2L-R), 5′-CGGCGTATTCTTCTCGGACT-3′; and 6-FAM-labeled probe, 5′-GCCTAGGAAGCGCTCCGGCG-3′.

The qPCR assay was performed using Luna® Universal Probe qPCR Master Mix (New England Biolabs, M3004) in a total reaction volume of 20 μL. Amplification was performed on a 7500 Fast Real-Time PCR System (Applied Biosystems, CA, USA). The thermal cycling conditions consisted of an initial denaturation at 95°C for 60 seconds, followed by 45 cycles of 95°C for 15 seconds and 60°C for 30 seconds, with fluorescence acquisition during the latter step.

The resulting qPCR amplicon was subjected to bidirectional Sanger sequencing. After trimming low-quality regions, the obtained sequences were analyzed using BLASTn to assess their consistency with the parapoxvirus *B2L* target.

## RESULTS

### Progression of Disease and Treatment

The patient was a 55-year-old male farmer who sustained an untreated scratch to his left ring finger from a sheep's tooth during slaughtering (day 0; Figure 1). On day 11, he developed fever (maximum temperature, 37.8°C), wound suppuration, chills, and rigors. He initially sought medical attention at a local hospital, where he received symptomatic wound care and oral moxifloxacin for 3 days, after which his body temperature decreased. On approximately day 14, following 3 days of oral moxifloxacin treatment, the patient experienced recurrent fever, with a maximum temperature of 38.5°C. Laboratory examinations at another hospital revealed a white blood cell count of 15.7 × 10^9^/L, an absolute neutrophil count of 11.36 × 10^9^/L, an absolute lymphocyte count of 2.5 × 10^9^/L, and a C-reactive protein level of 98.8 mg/L. He subsequently received intravenous levofloxacin for 3 days; however, intermittent fever persisted. After evaluation at the emergency surgical department and outpatient clinic of our hospital, he was admitted to the Infectious Diseases Department on day 17.

**Figure 1.**
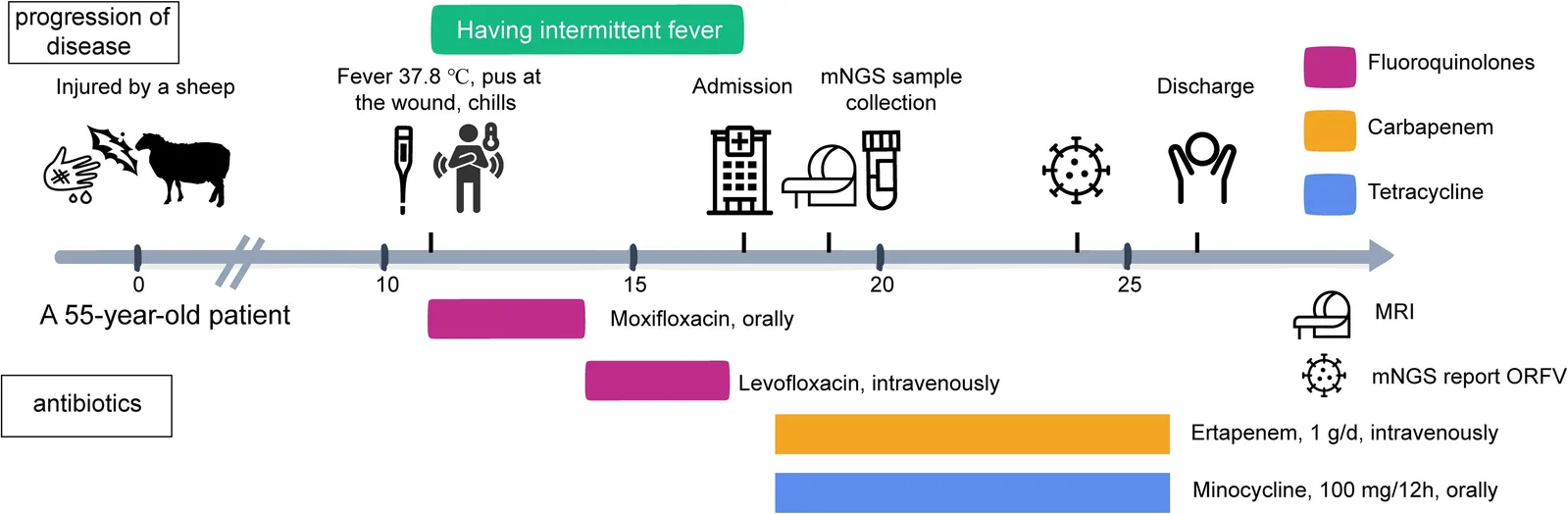
Timeline of disease progression and antimicrobial treatment. The day of the sheep-related injury was designated as day 0. The patient developed fever and wound suppuration on day 11 and received oral moxifloxacin for 3 days, followed by intravenous levofloxacin for 3 days because of recurrent fever. He was admitted on day 17. Intravenous ertapenem (1 g once daily) and oral minocycline (100 mg every 12 hours) were administered from day 18 through day 26. Contrast-enhanced MRI was performed on day 19, and the wound secretion specimen for mNGS was collected on day 20. The mNGS result identifying ORFV was reported on day 24. The patient received conservative local wound care during hospitalization and was discharged on day 26 with the wound nearly healed.

On admission, the patient had a body temperature of 37.8°C and a skin and soft tissue lesion involving the left ring finger (Figure 2*A*). By day 22 (5 days after admission), routine blood test results had returned to normal, the C-reactive protein (CRP) level had decreased to 10.1 mg/L, and the erythrocyte sedimentation rate had decreased to 31 mm/h. On day 26 (9 days after admission), the wound was nearly healed, with a dry surface and a small residual dark area (Figure 2*B*). No obvious purulent discharge or fluctuance was observed, and the patient was discharged. At follow-up on day 42 (25 days after admission), the wound had healed substantially (Figure 2*C*). During hospitalization, intravenous ertapenem (1 g once daily) and oral minocycline (100 mg every 12 hours) were administered from day 18 through day 26. Conservative wound management, including local wound cleansing and dressing changes, was also provided without surgical debridement.

**Figure 2.**
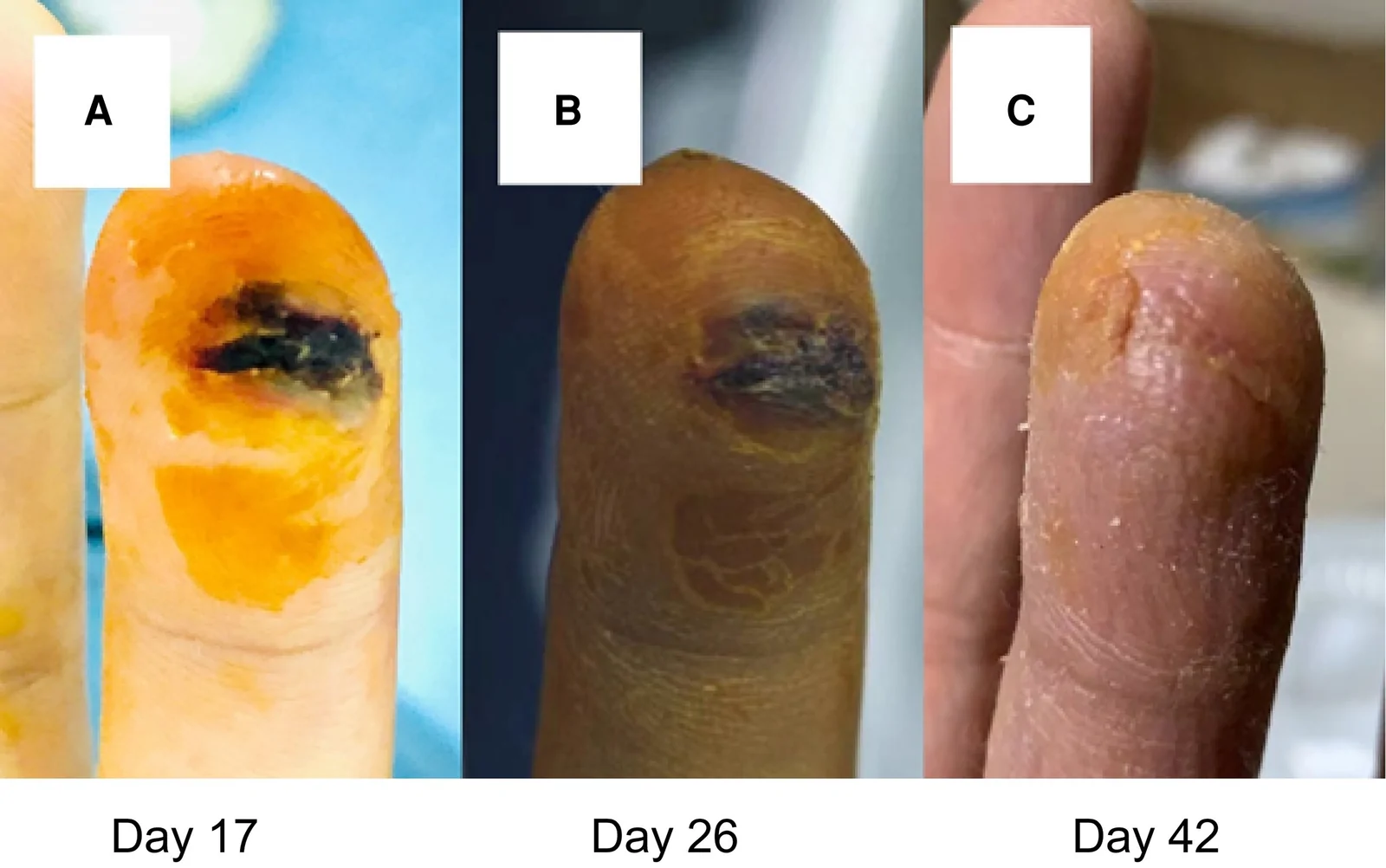
Clinical progression of the left ring finger lesion. *A*, The finger lesion on hospital admission (day 17). *B*, The wound at discharge (day 26), showing a dry surface and near-complete healing. *C*, Marked improvement of the lesion at follow-up (day 42, 25 days after admission).

### Routine Examination and Microbiological Examination

After admission, a physical examination showed a body temperature of 37.8°C, a pulse rate of 98 beats per minute, a respiration rate of 19 breaths per minute, and blood pressure of 127/77 mmHg. There was a skin and soft tissue infection of the left ring finger, and the rest of the examination found everything to be normal.

On admission, laboratory examinations revealed an elevated CRP level of 155.9 mg/L, a white blood cell count of 15.69 × 10^9^/L, a neutrophil percentage of 76.5%, an absolute neutrophil count of 12.0 × 10^9^/L, an absolute monocyte count of 1.29 × 10^9^/L, erythrocyte sedimentation rate at 67 mm/h, a prothrombin time of 13.1 seconds, a fibrinogen level of 824 mg/dL, alanine aminotransferase at 63 U/L, aspartate aminotransferase at 43 U/L, γ-glutamyltransferase at 75 U/L, alkaline phosphatase at 139 U/L, total bilirubin at 26.1 μmol/L, direct bilirubin at 10.5 μmol/L, glucose at 8.09 mmol/L, fecal occult blood positive, glycated hemoglobin at 6.2%, complement C4 at 0.45 g/L, rheumatoid factor at 14.3 IU/mL, and ferritin at 558 ng/mL. Additional laboratory findings included lymphocyte percentage at 14.7%, high-density lipoprotein cholesterol at 0.97 mmol/L, and sodium at 136.5 mmol/L. Contrast-enhanced MRI of the left hand was performed on day 19. Coronal contrast-enhanced T1-weighted water-only LAVA-Flex images demonstrated abnormal soft tissue enhancement surrounding the distal fourth finger (Figure 3). After admission, blood cultures were negative, and Gram staining, Ziehl–Neelsen staining, and bacterial and fungal cultures of the pus were all negative.

**Figure 3.**
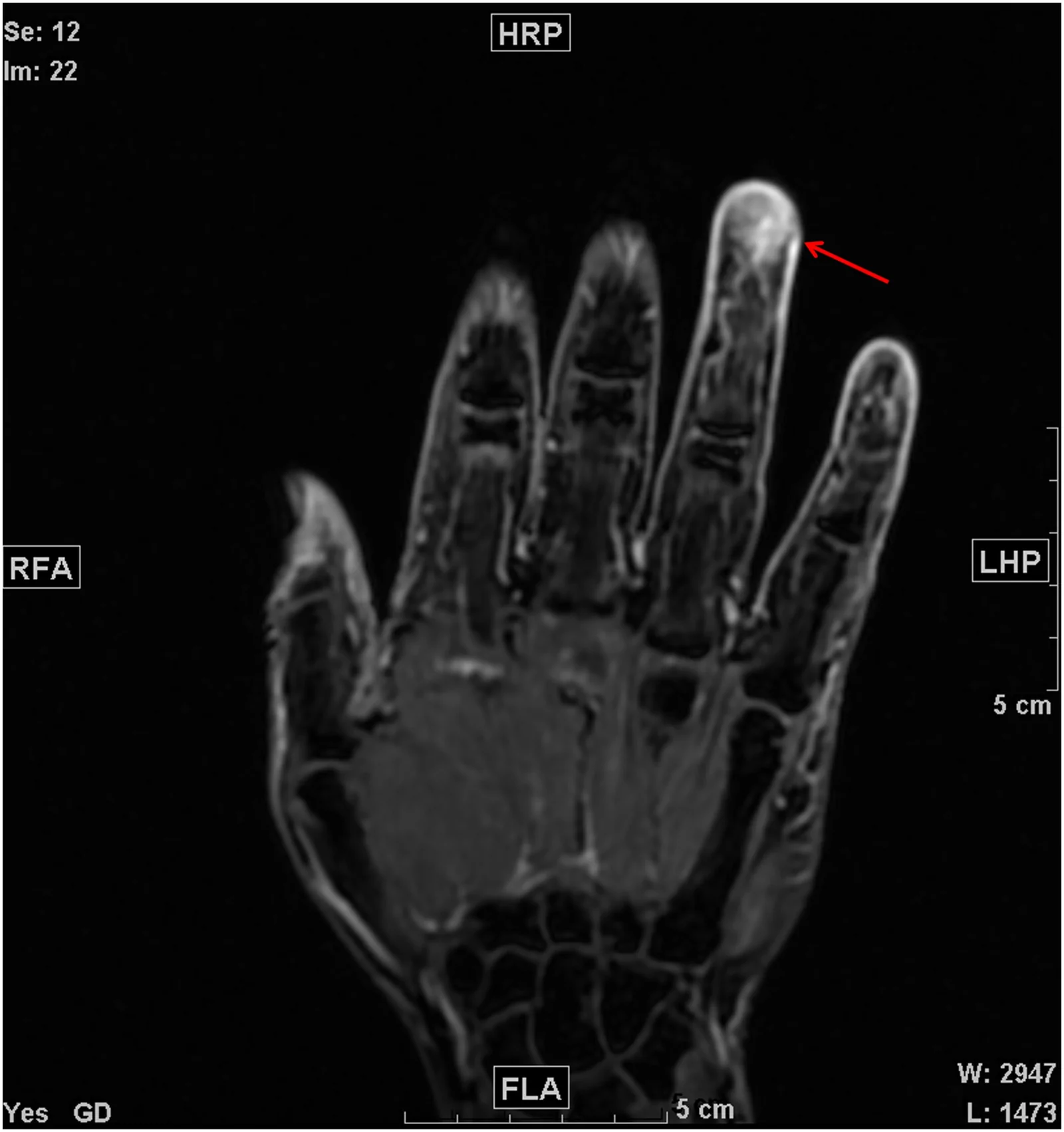
Contrast-enhanced magnetic resonance imaging of the left hand. Coronal contrast-enhanced T1-weighted water-only LAVA-Flex image showing abnormal soft tissue enhancement surrounding the distal phalanx of the left fourth finger (red arrow).

### Clinical Rationale for mNGS Testing and Identification of ORFV

Before mNGS testing, the patient presented with a persistent purulent finger lesion and recurrent fever following a sheep-related injury. The initial clinical assessment focused on a suspected post-traumatic soft tissue infection, while other bacterial and viral etiologies were also considered. Because the causative pathogen remained unidentified despite previous empirical antibiotic treatment and the initial diagnostic evaluation, mNGS was performed on a wound secretion specimen to investigate a broader range of potential infectious agents.

The wound secretion specimen was collected for mNGS on day 20, and the result identifying ORFV was reported on day 24. The mNGS analysis identified ORFV, with 724 unique sequence reads, 34.59% genome coverage, and an RPM ratio of 49.8, exceeding the predefined viral reporting threshold (RPM ratio ≥3). ORFV accounted for 100% of the relative abundance within the detected viral category (Figure 4). Together with the patient's sheep exposure history and characteristic cutaneous lesion, these findings supported ORFV as the principal etiological agent responsible for the skin lesion.

**Figure 4.**
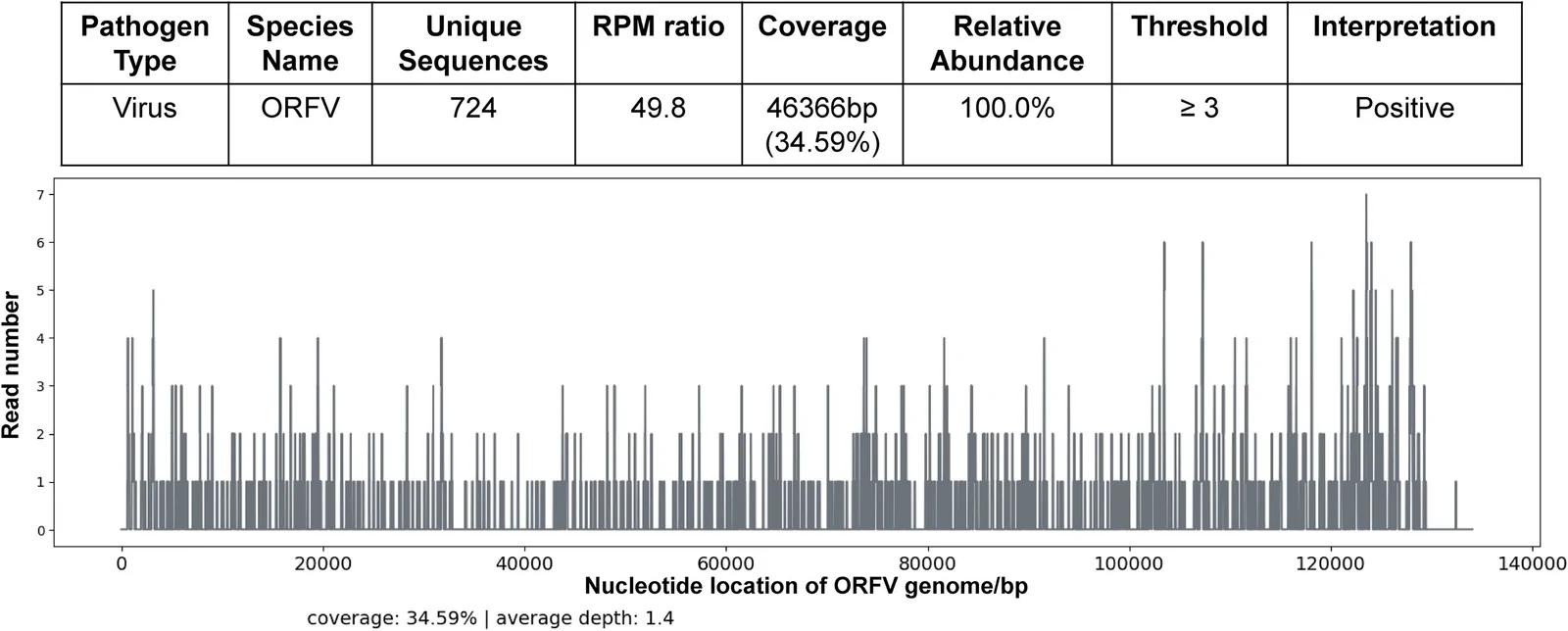
mNGS results identifying ORFV in the patient's wound secretion specimen. (Top) Summary table of the mNGS findings. The table details key metrics for ORFV, including the number of unique sequences (724), RPM ratio (49.8), genome coverage (46 366 bp, representing 34.59% of the reference genome), and relative abundance (100.0%). The detection was interpreted as positive based on the predefined threshold (RPM ratio≥3). (Bottom) The genome coverage plot of ORFV. The x-axis indicates the nucleotide location along the *orf* virus reference genome (in base pairs, bp), and the y-axis represents the number of mapped reads (read depth) at each position. The overall genome coverage is 34.59%, with an average sequencing depth of 1.4. Species-specific sequences: refers to the number of specific sequences of the microorganism detected at the species level. Coverage: refers to the percentage of the genome of the species that can be covered after all the detected sequences of the microorganism are mapped back to the genome. Relative abundance: refers to the distribution ratio of the sequences of a certain microbial species in the corresponding major categories of species (usually divided into 4 major categories: bacteria, fungi, viruses, and parasites) after removing the host sequences. RPM ratio: the ratio of the species-specific sequences of a species in the sample to the species-specific sequences of the corresponding species in the negative control.

Further analysis of bacterial and fungal communities identified additional microorganisms:

1. Bacterial detections

Among the bacterial taxa identified by mNGS, *Anaerococcus octavius* (RPM ratio, 50.4; genome coverage, 2.94%; relative abundance, 0.60%) and *Staphylococcus aureus* (RPM ratio, 16.2; genome coverage, 0.53%; relative abundance, 0.19%) exceeded the predefined bacterial reporting threshold (RPM ratio ≥10). Additional bacteria, including *Finegoldia magna*, *Peptoniphilus harei*, and *Streptococcus anginosus*, were detected at lower relative abundances (0.048%, 0.045%, and 0.017%, respectively) and were reported as possible findings according to the laboratory reporting criteria. However, detection above the reporting threshold does not necessarily indicate active bacterial infection.

2. Fungal detections

Among the fungal taxa detected by mNGS, *Malassezia restricta* accounted for 7930 sequence reads, with 6.52% genome coverage and a relative abundance of 97.9% within the fungal category. However, because the specimen was collected from a nonsterile wound site and conventional fungal cultures were negative, the clinical significance of this finding remained uncertain. Colonization or contamination could not be excluded, and the mNGS detection alone was insufficient to establish an active fungal infection.

Notably, no reads assigned to *Pseudomonas aeruginosa*, *Escherichia coli*, *Candida* spp., or *Aspergillus* spp. were detected (RPM ratio = 0). Additionally, no reads assigned to Epstein–Barr virus, cytomegalovirus, herpes simplex virus type 1, or the *Mycobacterium tuberculosis* complex were detected.

Although *A. octavius* and *S. aureus* exceeded the predefined bacterial reporting threshold, their clinical significance remained uncertain. The patient's fever, elevated inflammatory markers, and purulent wound following a sheep-related injury raised the possibility of concomitant bacterial superinfection. However, conventional bacterial cultures were negative, and no independent molecular confirmation of these bacteria was available. Therefore, bacterial colonization, contamination, or residual DNA from nonviable organisms following prior antibiotic exposure could not be excluded. Overall, ORFV was considered the principal etiological agent responsible for the characteristic cutaneous lesion, while a possible concomitant bacterial infection could not be definitively established or excluded.

### Molecular Validation of the mNGS Findings

To further evaluate the mNGS findings, *B2L*-targeted parapoxvirus qPCR was performed using the same wound secretion specimen analyzed by mNGS. The *B2L*-targeted parapoxvirus qPCR assay yielded a positive result. The approximately 78-bp PCR product was subsequently subjected to bidirectional Sanger sequencing, generating base-called reads of 47 and 54 bp in the forward and reverse directions, respectively (Supplementary Figure 1). BLASTn analysis of the reverse read identified matches to parapoxvirus *B2L* sequences, including ORFV, with 96.88% nucleotide identity, 57% query coverage, a bit score of 50.9, and an E-value of 0.014. The alignment covered 32 positions, with 31 identical positions and 1 gap. Comparable alignment statistics were also observed for a pseudocowpox virus *B2L* sequence (MG911971.1), highlighting the limited species-level discriminatory power of this short sequence. Given the short alignment length and sequence similarity among related parapoxviruses, the Sanger sequencing result was considered additional sequence-level support rather than independent species-specific confirmation. Together with the clinical presentation and sheep exposure history, the mNGS findings supported the diagnosis of ORFV infection.

## DISCUSSION

Although human ORFV infection occurs worldwide, it may not be routinely considered in the initial evaluation of skin and soft tissue infections. In the present case, the patient's sheep exposure history provided an important epidemiological clue, whereas the fever, purulent wound, and elevated inflammatory markers also raised concern for a bacterial wound infection. The persistence of symptoms despite prior antibiotic treatment and the absence of a pathogen identified by conventional microbiological testing complicated the initial etiological assessment.

In this clinical context, mNGS offered a broad-range diagnostic approach capable of detecting viral pathogens that would not be identified by routine bacterial and fungal cultures. However, the choice of diagnostic method should depend on the clinical suspicion and availability of targeted testing. When ORFV infection is strongly suspected based on animal exposure and characteristic lesions, targeted molecular testing may represent a more focused diagnostic approach. In contrast, mNGS may be particularly useful when the etiology remains uncertain or when a broader range of infectious agents needs to be investigated.

Human orf infection is generally diagnosed based on a history of relevant animal exposure and characteristic cutaneous lesions. When the diagnosis remains uncertain, additional laboratory methods, including targeted PCR, histopathological examination, and metagenomic sequencing, may help establish the infectious etiology. However, because human orf infection is generally self-limiting, molecular diagnosis should not be assumed to prevent disease progression or improve clinical outcomes. In the present case, mNGS contributed to the identification of ORFV, but the subsequent clinical improvement cannot be directly attributed to the diagnostic approach.

In this case, conventional microbiological examinations, including blood cultures, Gram staining, and bacterial and fungal cultures of wound secretions, did not identify an etiological agent. Although mNGS detected *A. octavius* and *S. aureus*, the clinical significance of these bacterial findings remained uncertain. The patient had received moxifloxacin and levofloxacin before admission, which may have reduced bacterial viability and contributed to negative culture results. In addition, the recovery of anaerobic bacteria, such as *A. octavius*, may be affected by specimen collection, transportation, and culture conditions. Nevertheless, these factors do not establish that the detected bacteria were responsible for an active infection. Because mNGS detects microbial nucleic acids regardless of organism viability, residual DNA from nonviable bacteria may persist following antimicrobial treatment. Therefore, the possibility of bacterial superinfection should be interpreted cautiously in conjunction with the clinical presentation and conventional microbiological findings. Importantly, the identification of ORFV by mNGS provided an etiological explanation for the characteristic skin lesion that was not addressed by routine bacterial and fungal culture methods.

In this case, *B2L*-targeted qPCR provided additional molecular evidence supporting the mNGS findings. However, because the assay was not independently validated for species-specific discrimination among parapoxviruses, a positive qPCR result alone could not establish ORFV infection. Furthermore, the short Sanger sequencing reads provided limited discriminatory power among closely related parapoxvirus species. A separate PCR targeting a longer *B2L* region, followed by bidirectional Sanger sequencing, could provide more informative sequence-level evidence and represents a potential approach for further validation. However, additional molecular testing was not feasible because the original clinical specimen was no longer available. Therefore, species-level identification of ORFV was primarily based on the mNGS results, interpreted in conjunction with the patient's sheep exposure history and characteristic cutaneous lesion. These findings highlight the complementary roles of broad-range metagenomic sequencing and targeted molecular testing in the diagnosis of uncommon viral infections.

Despite its broad pathogen detection capability [35–37], mNGS has important limitations when applied to nonsterile clinical specimens. Microbial nucleic acids detected in wound secretions may originate from active infection, colonization, environmental contamination, or nonviable microorganisms. Consequently, positive sequencing results do not necessarily indicate that the detected microorganisms are responsible for the patient's clinical manifestations. In the present case, although mNGS identified several bacterial and fungal microorganisms in addition to ORFV, their pathogenic significance could not be definitively established. Overinterpretation of these findings could potentially lead to unnecessary antibacterial or antifungal treatment. Therefore, mNGS results should be interpreted cautiously in conjunction with the clinical presentation, epidemiological history, conventional microbiological findings, and the pretest probability of infection.

The clinical value of mNGS depends on the diagnostic context and the questions being addressed. When ORFV infection is strongly suspected based on animal exposure and characteristic cutaneous lesions, targeted molecular testing may provide a more focused diagnostic approach. In contrast, mNGS may be particularly useful when the etiology remains uncertain, and a broader range of infectious agents needs to be investigated.

In the present case, mNGS identified ORFV, an infectious agent that had not been initially considered, and the finding was consistent with the patient's sheep exposure history and cutaneous presentation. Nevertheless, this single case does not establish the comparative diagnostic performance of mNGS or demonstrate that its use improves treatment outcomes. Rather, it illustrates the potential value of broad-range sequencing in selected diagnostically challenging cases, while emphasizing the importance of cautious interpretation and clinical correlation to avoid unnecessary treatment.

## Supplementary Material

ofag724_Supplementary_Data

## Supplementary Data

Supplementary materials are available at *Open Forum Infectious Diseases* online. Consisting of data provided by the authors to benefit the reader, the posted materials are not copyedited and are the sole responsibility of the authors, so questions or comments should be addressed to the corresponding author.
