# Supplementary material for: Metagenomic Next-Generation Sequencing as a Robust Diagnostic Approach for Human Orf Virus Infection Undetectable by Conventional Microbiological Methods
Source: Open Forum Infect Dis. 2026 Nov 13;13(11):ofag724. doi: 10.1093/ofid/ofag724 (PMC13759983; doi:10.1093/ofid/ofag724)
Supplement: Supplementary file 1 [file ofag724_supplementary_data.zip › Sup Fig1.pdf]

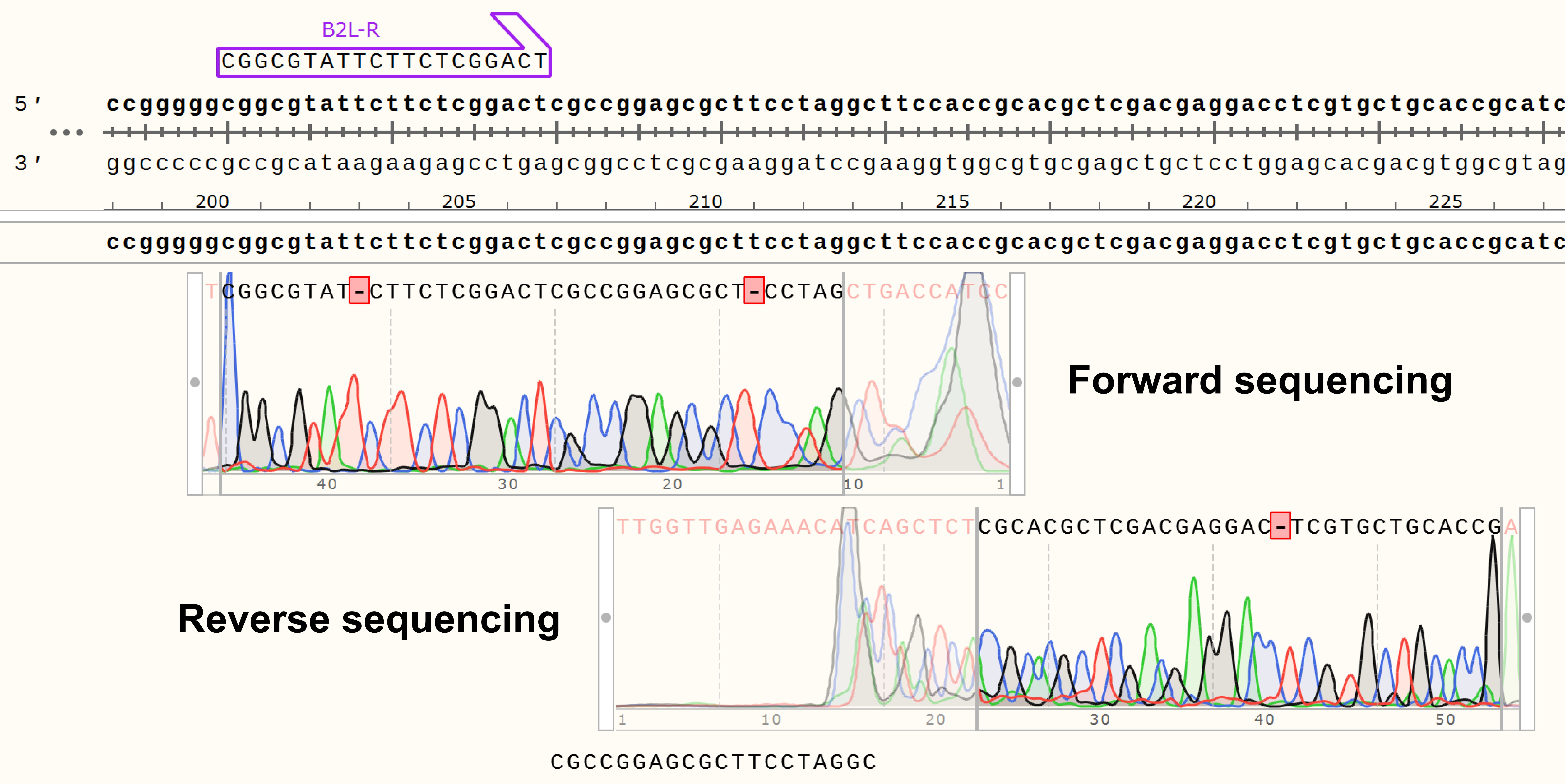
B2L-R
CGGCGTATTCTTCTCGGACT
5′
ccgggggcggcgtattcttctcggactcgccggagcgcttcctaggcttccaccgcacgctcgacgaggacctcgtgctgcaccgcatc
3′
ggccccgccgcataagaagagcctgagcggcctcgcgaaggatccgaaggtggcgtgcgagctgctcctggagcacgacgtggcgtag
200
205
210
215
220
225
ccgggggcggcgtattcttctcggactcgccggagcgcttcctaggcttccaccgcacgctcgacgaggacctcgtgctgcaccgcatc
TCGGCGTAT-CTTCTCGGACTCGCCGGAGCGCT-CCTAGCTGACCATCC
40
30
20
10
1
Forward sequencing
TTGGTTGAGAAACATCAGCTCTCGCACGCTCGACGAGGAC-TCGTGCTGCACCGA
1
10
20
30
40
50
Reverse sequencing
CGCCGGAGCGCTTCCTAGGC
