# Supplementary material for: Metagenomic Next-Generation Sequencing as a Robust Diagnostic Approach for Human Orf Virus Infection Undetectable by Conventional Microbiological Methods
Source: Open Forum Infect Dis. 2026 Nov 13;13(11):ofag724. doi: 10.1093/ofid/ofag724 (PMC13759983; doi:10.1093/ofid/ofag724)
Supplement: Supplementary file 1 [file ofag724_supplementary_data.zip › Supplementary Figure S1 caption.docx]

**Supplementary Figure S1. Bidirectional Sanger sequencing chromatograms of the B2L-targeted qPCR amplicon.**

Representative forward and reverse Sanger sequencing chromatograms of the approximately 78-bp B2L-targeted qPCR amplicon obtained from the wound secretion specimen. The sequences were aligned against the B2L gene reference sequence of orf virus strain ORFV/AH-FD/2016/China (GenBank accession no. KX951407.1). After quality trimming, the forward and reverse sequencing reads comprised 47 bp and 54 bp, respectively. The short sequencing reads and overlap with primer-binding regions limited their ability to distinguish ORFV from closely related parapoxviruses. Therefore, the Sanger sequencing results were interpreted as providing limited additional sequence-level support rather than independent species-specific confirmation of ORFV.
